# Paragangliome retropéritonéal: à propos d'un cas et revue de literature

**DOI:** 10.11604/pamj.2015.21.298.6564

**Published:** 2015-08-25

**Authors:** Yassine Fahmi, Tawfik Elabbasi, Driss Khaiz, Fatima Zahra Bensardi, Khaled Hattabi, Saad Berrada, Rachid Lefriyekh, Abdelaziz Fadil, Najib Zerouali

**Affiliations:** 1 Service des Urgences Chirurgicales Viscérales (P35), CHU Ibn Rochd, Casablanca, Maroc

**Keywords:** Tumeur rétropéritonéale, paragangliome, chirurgie, Retroperitoneal tumor, paraganglioma, surgery

## Abstract

Les paragangliomes rétropéritonéaux non fonctionnels sont des tumeurs rares. Ils sont définis comme des tumeurs chromaffines extra- surrénaliennes et représentent environ 1/5^ème^ des tumeurs chromaffines. Ils sont souvent asymptomatiques et peuvent atteindre des dimensions importantes. Nous rapportons l'observation d'une patiente âgée de 34 ans opérée pour une tumeur rétropéritonéale géante et dont l'examen anapath a conclu à un paragangliome. Les formes malignes, plus fréquentes que les formes bénignes, présentent un envahissement locorégional et métastasent tardivement. La prise en charge des paragangliomes doit être multidisciplinaire mais seul le traitement chirurgical est curatif. Il n'existe par contre pas de consensus sur l'utilité des thérapeutiques complémentaires qui peuvent néanmoins constituer un appoint à titre symptomatique. Les paragangliomes présentent un caractère génétique dans 25% des cas. Une enquête génétique doit être systématiquement proposée.

## Introduction

Les paragangliomes ou phéochromocytomes extra-surrénaliens, sont des tumeurs neuroendocrines développées aux dépend du système nerveux parasympathique [1]. Ils sont définis comme des tumeurs chromaffines extra-surrénaliennes et représentent environ 1/5^ème^ des tumeurs chromaffines. Les paragangliomes rétropéritonéaux non fonctionnels sont des tumeurs rares [2, 3] et sont moins fréquentes que les autres localisations (tête, cou) [2]. Ils sont souvent asymptomatiques et peuvent atteindre des dimensions importantes. La prise en charge des paragangliomes doit être multidisciplinaire mais seul le traitement chirurgical est curatif.

## Patient et observation

**Figure 1:**
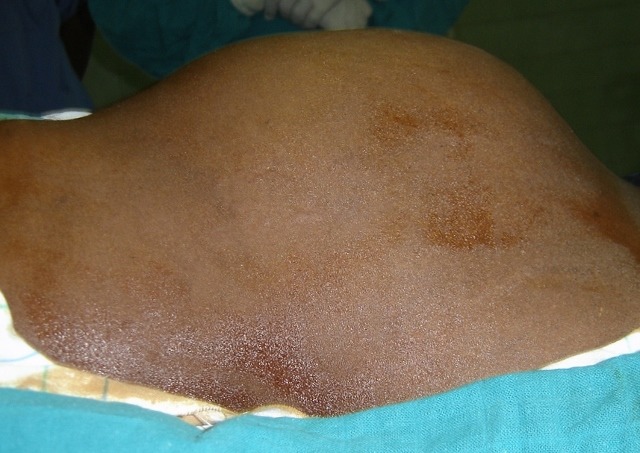
Masse abdominale allant de l'hypochondre droit à la fosse iliaque droite

Il s'agit d'une patiente, âgée de 34 ans, mère de 4 enfants sans antécédents pathologiques notables opérée deux mois avant son admission au service, pour une tumeur abdominale adhérente au gros vaisseaux (aller retour) à Nouagchot en Mauritanie et pour laquelle elle nous a été adressé. A l'admission la patiente était consciente, en bon état général, IMC: 21 kg/m2. L'examen abdominal note une cicatrice de laparotomie médiane à cheval sur l'ombilic et la présence d'une masse allant de l'hypochondre droit à la fosse iliaque droite, dure, battante, fixe par rapport au plan profond de 18cm/14cm (Figure 1). Le bilan biologique était normale du même que l'ECG et la radio de thorax

**Figure 2:**
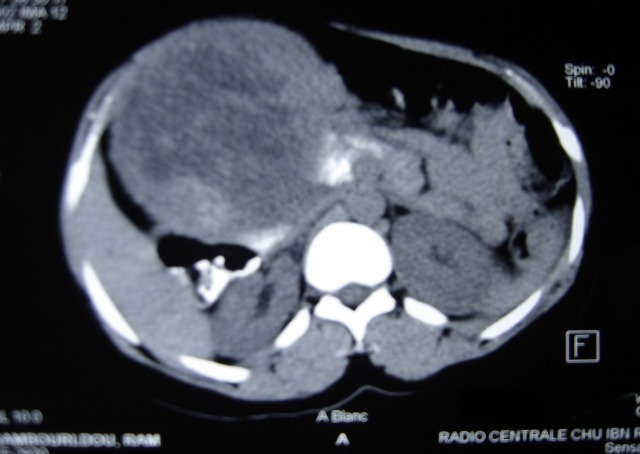
Volumineuse tumeur péritonéale du flanc droit mesurant 14 x 12,5cm d'origine mésenchymateuse ou stromale à partir de la VCI très probablement

**Figure 3:**
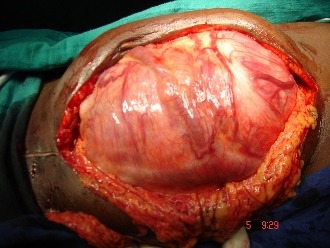
Tumeur dure rétro péritonéale hyper vascularisée

**Figure 4:**
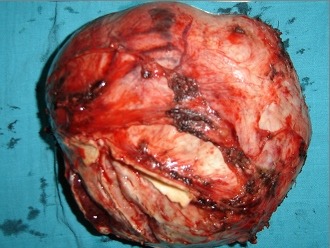
Tumeur dure rétro péritonéale hyper vascularisée, adhérente au 3ème duodénum, englobant la VCI et l'aorte abdominale sous rénale jusqu’à leur bifurcation

**Figure 5:**
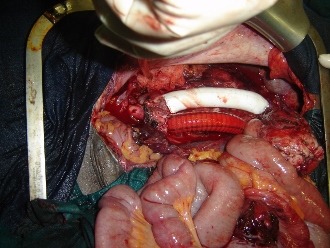
Double prothèse de l'aorte et de la VCI

La TDM abdomino thoracique (Figure 2) note une volumineuse tumeur péritonéale du flanc droit mesurant 14 x 12,5cm d'origine mésenchymateuse ou stromale à partir de la VCI très probablement avec absence de métastase pulmonaire. Echo doppler de la VCI notait sa non visualisation au contact de la masse, et soit qu'elle est thrombosée ou laminée. On décide de l'explorer après une coordination entre chirurgien viscéraliste, chirurgien cardiovasculaire et anesthésiste réanimateur. la patiente a été explorée avec reprise de l'ancienne cicatrice de la laparotomie médiane et découverte d’ une tumeur dure rétro péritonéale hyper vascularisée (Figure 3), adhérente au 3ème duodénum, englobant la VCI et l'aorte abdominale sous rénale jusqu’à leur bifurcation (Figure 4), réalisation d'une exérèse totale de la tumeur emportant l'aorte abdominale et la VCI en sous rénale jusqu’à leur bifurcation et on procède à une double prothèse de l'aorte et de la VCI (Figure 5) et suture de l'effraction duodénale, avec drainage par deux drains de Redon un en rétro péritonéale et l'autre dans le Douglas.

La patiente fut hospitalisée en réanimation, à J3 post opératoire, on a noté l’ issue de la bile par les orifices de drainage; une reprise a été décidée montrant une perforation du 3^ème^ duodénum en réalisant une résection duodénale (au niveau du genus inférieur) avec anastomose termino-terminale, cholécystectomie avec drainage de la VBP par drain de Kher, dérivation gastro jéjunale par anastomose gastro jéjunale après exclusion définitive du duodénum par pince TA 55 au niveau du pylore puis drainage par 2 lames de Delbet en pré et rétro anastomotique. Dans les suites post opératoire: A J9: thrombose veineuse profonde du membre inférieure gauche; Echo doppler: thrombose étendue de la veine iliaque externe et la veine iliaque interne gauche; Angioscanner abdominale: Processus tissulaire rétro péritonéal englobant l'aorte et la veine cave inférieure avec envahissement du psoas et uretéro hydronéphrose d'amont; Thrombose de la VCI étendue à la veine iliaque externe droite et la veine fémorale commune gauche.

La patiente est mise sous heparine sodique à dose hypo coagulante 400U/kg/j Puis sous AVK (1/2 cp de Sintrom/j), lovenox 0,6/j, aspegic 250; surveillance par TA. INR et NFS. Puis arrêt du Lovenox et poursuite de la prise du Sintrom ¾ cp, avec suivi par un cardiologue. L'examen anatomopathologique de la pièce opératoire a conclu à un aspect morphologique et immunohistochimique en faveur d'un paragangliome.

## Discussion

Les tumeurs rétropéritonéales primitives (TRP) de l'adulte sont le plus souvent malignes. Leur prévalence représente moins de 1% des tumeurs. Leur découverte est souvent tardive alors qu'elles atteignent une très grande taille. L'atteinte tumorale du rétropéritoine est hétérogène. Elle est composée de 80% de lésions malignes dont plus de la moitié sont des sarcomes. Le pronostic de ces TRP dépend de leur grade histologique et de la résection chirurgicale complète. Le risque de récidive locale est supérieur au risque métastatique.

Les TRP sont classées en fonction de leur origine embryonnaire, ainsi les paragangliomes sont des tumeurs ectodermiques d'origine neurogène et sont définis comme des tumeurs chromaffines extra-surrénaliennes. Les phéochromocytomes extra-surrénaliens ou paragangliomes représentent environ 1/5^ème^ des tumeurs chromaffines et sont une entité rare et sont sécrétant dans 60% des cas [4]. Embryologiquement, les cellules chromaffines s’étendent de la base du crâne à l’épididyme. La plupart involuent; et seules persistent celles de la surrénale et des chaînes ganglionnaires rétropéritonéales et thoraciques. Les phéochromocytomes extra-surrénaliens ou paragangliomes comptent pour 18% des tumeurs chromaffines. Ils surviennent à un âge plus précoce, 10 à 30 ans mais plus fréquents chez l'adulte jeune [4]. Ils sont multifocaux dans 15 à 24% des cas. Ils sont beaucoup plus souvent malins que les phéochromocytomes intra-surrénaliens environ 40% contre 10%. Les formes malignes surviennent plus précocement que les formes bénignes et se caractérisent par la survenue d'un envahissement locale (type lymphatique) ou à distance (envahissement du poumon, os, foie) dans 30% des cas [3, 4]. Ils sécrètent de manière prédominante de la noradrénaline, souvent exclusivement. Leur origine est génétique, ce qui correspond bien à leur multicentricité et les récidives métachrones dans d'autres sites. Seul 1% des paragangliomes de la tête et du cou sécrètent des catécholamines alors que ceux de siège thoracique, abdominal, rétro-péritonéal ou pelvien sécrètent plus souvent [5]. Onze cas de paragangliomes pulmonaires ont été publiés. Enfin, seulement deux cas de paragangliomes ou de phéochromocytomes primitivement intra-hépatiques ont été décrits. Le phéochromocytome n'apparaît pas seulement comme une tumeur de la glande surrénale, mais plutôt une maladie du système chromaffine. L'origine génétique est évoquée plus lorsque les tumeurs sont multiples, tandis que la malignité est d'autant plus élevée lorsque la tumeur est ectopique.

Les formes rétropéritonéales sont le plus souvent isolées [3, 4]. Ils sont associés parfois à d'autres pathologies, notamment la triade de Carney [6], avec la néoplasie endocrinienne multiple type 2 [3] et neurofibromatose de type 1. Les paragangliomes rétropéritonéaux non fonctionnels se caractérisent par leur aspect asymptomatique (absence d'HTA) et des taux normaux de catécholamines sanguins et urinaires [3] et une latence clinique le plus souvent [4], parfois des signes non spécifiques sont retrouvés [4]. Le diagnostic positif préopératoire est biologique [7]. L’échographie abdominale note une masse ovalaire, solide bien limitée avec de nombreuses formations centrales d'allure kystique [3, 6]. La TDM abdominale montre les caractères de la tumeur notamment son siége rétropéritonéal, sa taille, son caractère unique ou multiple avec recherche d'envahissement locorégional et à distance, l'aspect le plus souvent retrouvé est celui d'une masse solide ronde ou ovale, homogène, mais pouvant être kystique ou nécrosée en son centre ou calcifiée [3, 8, 9] alors que l'IRM est l'examen de choix pour le diagnostic et le bilan morphologique des lésions, pour la TDM elle est équivalent de l'IRM pour le bilan morphologique mais toute fois son efficacité est moindre. La scintigraphie à la méta- iodo- bénzyl-guanidine a peu d'intérêt en préopératoire, mais occupe une place majeure dans la surveillance opératoire. Le diagnostic de certitude est histologique [3, 4], avec un aspect d’énorme tumeur arrondi, encapsulée de consistance ferme, élastique, très vascularisée, mais c'est l'immunohistochimie qui permet d'affirmer le diagnostic, par contre il n'existe aucun critère histologique permettant de distinguer entre la bénignité et la malignité de la tumeur [8]. Une bonne prise en charge du paragangliome passe par un bilan morphologique précis, vue la complexité vasculaire de ces tumeurs d'où l'intérêt de se baser sur l'angioscanner. La chirurgie radicale constitue la base du traitement avec résection radicale dans 75% des cas [4].

Le choix du procédé chirurgical entre la voie conventionnelle et caelioscopique reste très controversé vue les effets indésirables de la laparoscopie qui sont indéniables Les thérapies complémentaires, type chimiothérapie, radiothérapie externe pourraient trouver leur place dans les formes métastatiques avec une réponse positive dans environ 50% des cas, mais sans influencer le pronostic de manière significative, seul l'exérèse chirurgicale permet une amélioration significative, avec un taux de survie sans récidive de 75% à 5 ans et de 45% à 10 ans [4] La moyenne de survie est de l'ordre de 3 ans dans les formes métastatiques et de 4 ans en cas d'exérèse incomplète [4].

## Conclusion

Les paragangliomes ou phéochromocytomes extra- surrénaliens, sont des tumeurs neuroendocrines développées aux dépend du système nerveux parasympathique Les paragangliome rétropéritonéaux non fonctionnels sont des tumeurs rares. Ils sont souvent asymptomatiques et peuvent atteindre des dimensions importantes La prise en charge des paragangliomes doit être multidisciplinaire mais seul le traitement chirurgical est curatif Les thérapies complémentaires, type chimiothérapie, radiothérapie externe pourraient trouver leur place dans les formes métastatiques mais sans influencer le pronostic de manière significative.
